# Nutritional Status and Ergogenic Aids in Performance During Exercise and Sports

**DOI:** 10.3390/nu17071224

**Published:** 2025-03-31

**Authors:** David Varillas-Delgado

**Affiliations:** 1 Exercise and Sport Science, Faculty of Health Sciences, Universidad Francisco de Vitoria, 28223 Pozuelo, Spain; david.varillas@ufv.es or d.varillas@sportnomics.es; 2 SPORTNOMICS S.L., 28922 Madrid, Spain

The interaction between nutritional status, ergogenic aids, and athletic performance has long been a central focus in sports science. By integrating findings from five significant studies, this editorial aims to provide a comprehensive perspective on the current landscape of nutritional interventions and their impact on exercise and sports performance. However, the practical applicability of most findings in this field remains limited, requiring careful consideration of several factors, especially in nutrition, supplementation strategies, and gene–gene interactions. Future research should prioritize larger sample sizes and include replication cohorts to enhance reliability and generalizability associations in sports science. This Special Issue, entitled “*Nutritional Status and Ergogenic Aids in Performance during Exercise and Sports*”, compiles four recent advances and one systematic review that explore the identified gaps in knowledge and outlines future research directions.

One of the key studies in this issue, conducted by Varillas-Delgado [1], investigates the association between genetic profiles in muscle performance and the effectiveness of creatine supplementation in professional football players. The polymorphisms in the angiotensin-converting enzyme (*ACE*) I/D (rs4646994), alpha-actinin 3 (*ACTN3*) c.1729C>T (rs1815739), adenosine monophosphate deaminase 1 (*AMPD1*) c.34C>T (rs17602729), muscle-specific creatine kinase (*CKM*) c.*800A>G (rs8111989), and myosin light-chain kinase (*MLCK*) [c.49C>T (rs2700352) and c.37885C>A (rs28497577)] were evaluated in 161 professional football players. Previous studies have identified associations between specific polymorphisms and muscle and tendon injury susceptibility, as well as their potential role in talent identification in sports [2,3,4]. These genetic variants have been linked to key physiological traits, including muscle performance, recovery capacity, and injury resistance [5,6,7,8]. The investigation highlights the role of these polymorphisms, particularly the *AMPD1* rs17602729, in modulating muscle mass gains and injury prevention by using total genotype score (TGS). The results indicate that individuals with the CC genotype and C allele in *AMPD1* exhibit a greater increase in muscle mass and body mass index (BMI) following creatine supplementation, whereas those with a lower TGS cut-off point face a higher risk of non-contact muscle injuries during the football season [1]. This investigation reinforces the importance of precise nutrition, where genetic profiling could help tailor supplementation strategies for enhanced performance and injury mitigation.

Ewell et al. [9], in a randomized, double-blind, placebo-controlled cross-over pilot study, examined the effects of acute oral lactate supplementation on cycling performance in recreational exercisers. Participants ingested either a placebo or a lactate supplement (19 ± 1 mg/kg body mass) before undergoing incremental exercise until fatigue and a 20 min time trial on a cycle ergometer. Acute oral lactate supplementation has shown potential benefits in enhancing high-intensity cycling performance by increasing blood bicarbonate levels and possibly improving time until exhaustion. However, the results are inconsistent, and further research is needed to fully understand its implications and optimize its use in different cycling contexts. Key studies [10,11,12] provide insights into these effects, highlighting both the potential and limitations of lactate as an ergogenic aid. In Ewell et al.’s [9] investigation, no significant differences were found between the lactate supplement and placebo in the peak oxygen uptake (VO_2_peak), ventilatory threshold, or work rate at the lactate threshold during incremental exercise. However, during the 20 min time trial, lactate supplementation resulted in a modest but statistically significant 4% increase in the average work rate compared to the placebo (*p* = 0.02). Importantly, this enhancement occurred without affecting heart rate or ratings of perceived exertion, suggesting its ergogenic benefit without additional physiological strain. These findings indicate that while acute oral lactate supplementation may not alter physiological responses during incremental exercise, it can modestly enhance performance during short-duration, high-intensity efforts. This investigation contributes to the growing body of literature exploring nutritional strategies to optimize athletic performance and suggests the potential application of lactate supplementation in endurance sports.

Devrim-Lanpir et al. [13] explored the effects of acute citrulline malate (CM) supplementation on CrossFit^®^ performance and cardiovascular responses in a randomized, double-blind, placebo-controlled, cross-over study. Previous systematic reviews critically evaluated the effects of citrulline malate supplementation on exercise performance. They highlight that citrulline malate may enhance endurance and reduce muscle soreness, leading to improved overall athletic performance. These reviews indicate that this supplementation is particularly effective for high-intensity exercise and in repeated bouts, suggesting the potential benefits for athletes seeking to optimize their training and recovery. However, further research on this topic is also needed to establish the optimal dosages and long-term effects of citrulline malate on various populations [14,15]. The study of Devrim-Lanpir et al. [13] found no significant difference in the number of rounds completed between the CM and placebo conditions. However, CM supplementation resulted in a significant reduction in heart rate during the workout and a shorter post-exercise recovery time, indicating improved cardiovascular efficiency, contrary to previous results [16,17,18]. These findings suggest a heightened cardiovascular response, which may have implications for endurance performance. However, the mixed results reported across the literature regarding CM efficacy indicate the necessity of additional research, particularly regarding the optimal dosages, exercise modalities, and individual variability in responses.

The study of Abassi et al. [19] demonstrates that high-intensity interval training (HIIT) significantly reduces liver enzyme levels (ALT and AST) and improves the biomarkers associated with metabolic dysfunction-associated steatosis liver disease (MASLD) in overweight/obese adolescent girls. The findings indicate that HIIT is an effective intervention for enhancing hepatic and metabolic health in this population, highlighting its potential in preventing obesity-related complications, as previously shown [20,21]. The results of this study align with previous findings that support HIIT as an effective tool for reducing visceral fat and improving metabolic markers [22,23,24]. However, this investigation advances the field by incorporating a gender-specific focus and a more detailed assessment of hepatic biomarkers. The study suggests that HIIT could be a promising therapeutic strategy for managing MASLD in youth. However, future research could explore the long-term effects of HIIT, compare different training protocols (duration, frequency, and intensity), and evaluate its impact on more diverse populations, including different ethnic groups and developmental stages.

There is one high-quality review in this Special Issue. A broader perspective on dietary supplementation is provided by Harlow et al. [25], examining randomized and quasi-experimental controlled trials to assess the impact of dietary supplements on physical performance and recovery in active-duty military personnel. Its key findings indicate that supplements such as caffeine, branched-chain amino acids (BCAAs), creatine, and β-alanine significantly enhance physical performance, endurance, and muscle recovery. However, the results presented in this review vary depending on the type of supplement, dosage, and operational context, aspects that should encompass the individual responses of each subject, as demonstrated in the study by Varillas-Delgado [1], which explores the role of genetic profiles in responses to creatine among professional football players. Gaps in the knowledge include the lack of long-term studies on the safety and efficacy of these supplements, as well as the need to investigate their effects under extreme conditions (altitude, heat, and stress) or genetic associations [26]. Additionally, more evidence is required on the interactions between supplements and habitual diets. Future research should focus on standardized protocols and more diverse populations to optimize recommendations for military settings.

Although the field of nutrition and ergogenic aids in performance during exercise is fascinating, the lack of research integrating sports science, nutrition, and clinical practice will be pivotal in driving innovation (or not) in this field. Such collaborative efforts remedying this lack would pave the way for evidence-based, personalized strategies that enhance athletic performance while ensuring the health and well-being of athletes across different domains. By fostering the continued exploration of and discussions on this topic, this Special Issue serves as a catalyst for further advancements in nutritional interventions in sports performance.
